# Grey Filter Contact Lens as Therapeutic Option for Acquired Reduced Binocular Visual Performance

**DOI:** 10.22599/bioj.169

**Published:** 2021-03-29

**Authors:** Hans van Vliet, Hinke Marijke Jellema, Carla Nieuwendaal, Ruthie Lapid-Gortzak, Frans Riemslag, Ivanka van der Meulen

**Affiliations:** 1 Amsterdam UMC: Oculenti contact lenses, NL; 2 Amsterdam UMC, NL; 3 Bartiméus, NL

**Keywords:** acquired unilateral vision loss, binocular vision, grey filter contact lens, intractable diplopia, peripheral field of vision

## Abstract

Disturbing binocular problems can be too complex to be treated in such a way that comfortable binocular single vision is restored. The grey filter contact lens could offer a safe and clinically useful way to help these patients.

**Background:**

In unilateral acquired reduced visual performance or intractable diplopia the binocular performance often is less than the performance of the better eye, possibly leading to complaints of binocular visual functioning. The hypothesis is to use a grey filter contact lens on the affected eye to obtain more binocular visual comfort. The grey filter changes the binocular central visual image in the brain through delaying the image of the affected eye and has minimal effect on the peripheral vision. The purpose of this study was to evaluate the effect of the grey filter contact lens on the reduction of patients’ binocular complaints in daily life.

**Methods:**

In 19 consecutive patients with unilateral acquired reduced visual performance or intractable diplopia a grey filter contact lens was fitted. The contact lens was chosen from six available filters with different transmissions, based on patient preference. The chosen filter contact lens was fitted according to the normal practice of contact lens fitting.

**Results:**

The results of 18 patients are reported, one patient was lost to follow-up. Twelve patients (67%) reported good results when wearing the grey filter contact lens. Five patients (28%) discontinued wear of the grey filter contact lens because their binocular visual complaints disappeared during filter contact lens wear and remained absent after contact lens wear was terminated.

**Conclusion:**

The grey filter contact lens is a clinically useful, safe, and easily reversible treatment option for patients with binocular visual complaints due to an acquired monocular reduction in visual quality.

## Introduction

Every clinician in the optometry or orthoptic department is familiar with the complex patient suffering from visual disturbance. This acquired diagnosed reduced binocular performance is caused by monocular visual acuity loss due to pathology or trauma. Another group of patients with acquired reduced binocular visual performance has no monocular visual acuity loss (e.g., loss of sensory fusion or pathological changes in the optic nerve). In the latter group of patients, the less dominant image disturbs the more dominant image of the other eye. Both categories of patients suffer from their eye condition in daily life. Binocular visual performance is generally better than monocular visual performance (Blake, Sloane & Fox 1981). However, several factors can hamper this binocular performance (Gagnon & Kline 2003; Jimenez, Ponce & Anera 2004; Kattan et al. 2016; Lema & Blake 1977; Pineles et al. 2011). When unilateral reduced visual acuity is present, binocular visual acuity can be less than the visual acuity of the better eye, which is called binocular inhibition (Pineles et al. 2013). A diminished binocular vision and stereopsis affects day to day visuomotor skills like walking, driving a car, pouring coffee, end-phase prehension, and so forth (Buckley et al. 2010; Buckley 2015; Grant & Moseley 2011). Previous studies have suggested worsening of binocular visual function after acquired unilateral reduced visual performance (Pardhan & Gonzalez-Alvarez 2005; VU et al. 2005). Patients with acquired unilateral reduced visual performance can be divided in two groups. The first group consists of participants with a disease in one eye, leading to monocular deteriorated visual acuity and thus an inter-ocular difference in visual performance. The second group consists of participants with a disease in both eyes (for example, age-related macular degeneration), which can result in monocular unequal diminished visual acuity or performance. An event could also result in patients with intractable diplopia without deteriorated visual acuity in either eye but with a noticeable disturbing image of the less dominant eye (e.g., loss of sensory fusion).

Because of the diversity of causes, there is not much knowledge about the incidence of these visual problems. There is no study about overall incidence of acquired reduced binocular visual performance due to pathologies with monocular visual acuity loss. An incidence of intractable diplopia of 53 cases per year in the UK is reported (Newsham, O’Connor & Harrad 2018). Other studies showed three patients with intractable diplopia after 424 adults were operated for strabismus (Kushner 2002) and 32 patients out of 239 consecutive patients with brain damage (Fowler et al. 1996).

An option to improve visual performance is to induce a diffuse defocus in the affected or less dominant eye, to hamper the disturbing image. This can be achieved by a change in the refractive power, Bangerter filter, or opaque tape (De Pool et al. 2005; Iacobucci, Furr & Archer 2009; Rutstein 2010). Bangerter filter is a thin filter in front of a spectacle lens, with a density to degrade the image of the affected eye. This still allows peripheral vision, but it is degraded (Rutstein 2010).

**Photo 1:**
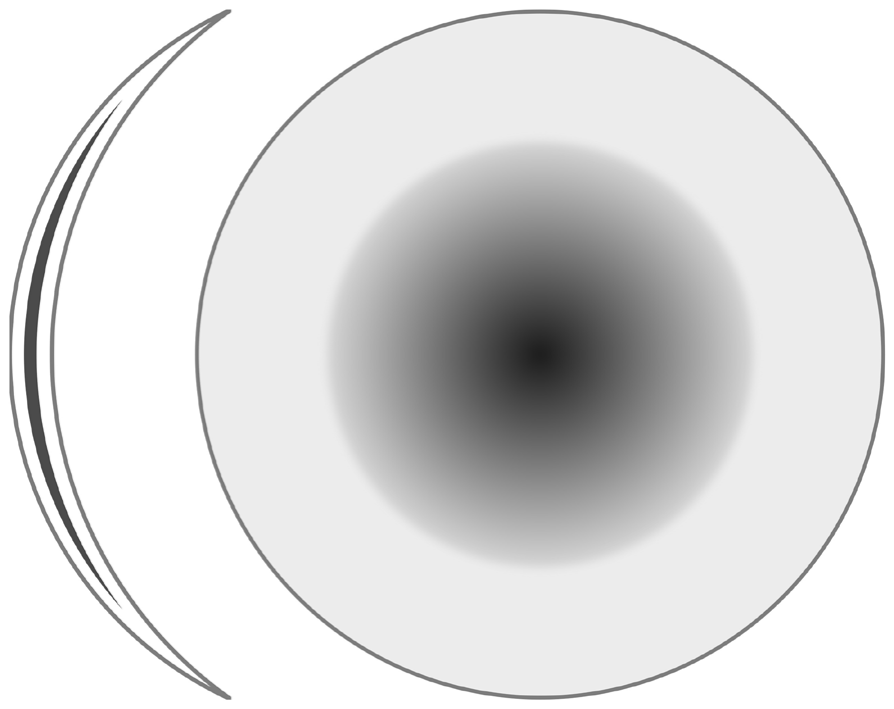
Scotogenic contact lens.

It is also possible to attempt to improve visual performance by completely eliminating the disturbing image from the affected or less dominant eye by an eye-patch, opaque tape, or an occluding contact lens, and rarely with an occluding intraocular lens (IOL) or a black corneal tattoo in the center of the cornea (Astin 1998; Newsham, O’Connor & Harrad 2018; Shonibare & Lochhead 2014). However, this limits the field of vision to that of the unaffected eye and can thus change everyday tasks. A scotogenic contact lens (***Photo 1***) may solve this problem, as it is a soft contact lens with an occluding centre and gradually increasing transmission towards the periphery, which leaves the peripheral field of vision unaffected (Robert et al. 2015).

**Photo 2:**
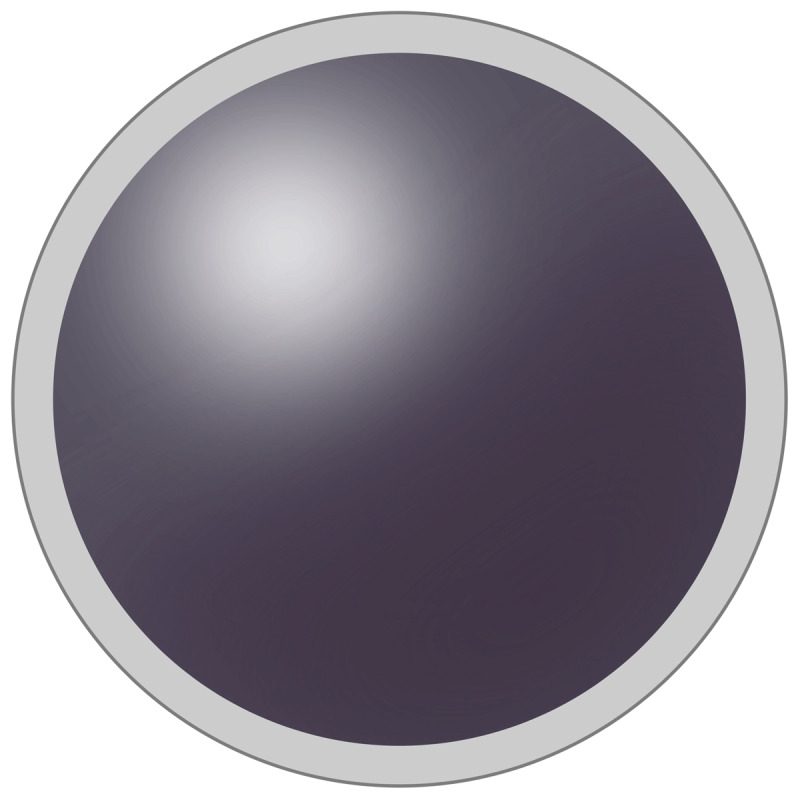
Grey filter contact lens.

We have performed this study with the grey filter contact lens, which differs from the scotogenic contact lens by not creating a central scotoma. In contrast to the scotogenic contact lens, the centre of the grey filter contact lens is not black, does not occlude. Experimentally using Neutral Density (ND) filters can achieve a delay in VEP latency (Heravian-Shandiz & Jenkins 1991). A grey filter, like an ND filter and unlike any other color filter, reduces the intensity of all wavelengths equally. A contact lens with grey filter has coverage of all light entering the eye, which cannot be achieved by grey spectacle glasses. The grey filter contact lens (***Photo 2***) is fully light transmittable, and the density of the grey filter, constant over the diameter of the filter in the contact lens, changes the delay in time of both central and peripheral stimuli. For instance, in patients with multiple sclerosis the image or stimulus of the eyes could not be conducted equally in time to the brain. The stimulus of the most affected eye is delayed compared to the stimulus of the least affected eye with recordings of visual evoked potentials (VEP). A short delay of the VEP latency (less than 40 msec.) can inhibit the response to the second stimulus completely (Riemslag, Spekreijse & Wessem 1985). This delayed stimulus of the most affected eye is called the second stimulus. If the response to a disturbing image of the eye can be delayed, but less than 40 msec., the inhibition of this image only occurs in the central visual field. As a result, the central visual performance is exclusively formed by the image of the unaffected eye. However, the peripheral visual field remains intact and both the affected and the unaffected eye continue to contribute to the peripheral visual field.

The aim of this study was to evaluate patients’ response to the effect of grey filter contact lenses on the binocular complaints of patients with acquired reduced binocular visual performance. The effect of grey filter contact lenses on acquired reduced binocular performance by changing the binocular central visual image in the brain through delaying the image of the affected eye has not been reported before.

## Methods

This is a retrospective cohort study of patients who were referred by optometrists, orthoptists, or ophthalmologists from secondary care clinics in and around Amsterdam to a tertiary care clinic to fit a grey filter contact lens. The study adhered to the tenets of the Declaration of Helsinki and received approval from the Medical Ethical Committee of the Academic Medical Centre in Amsterdam, the Netherlands. Due to the retrospective nature of this study no individual informed consent was necessary. The data were collected clinically between April 2012 and May 2017 and gathered for analysis from patient files. All subjects were thoroughly examined by the referring professional, and all other solutions to diminish complaints of binocular reduced performance were excluded. The patients wore their best refractive correction.

All patients had a complete evaluation that included history, visual acuity, refraction, and slit lamp examination. If an asymmetrical visual acuity is present, the eye with the worse visual acuity is called the affected eye in this study. If no asymmetrical visual acuity is present but there is a disturbing image in one eye, the eye with the disturbing image is the affected or less dominant eye in this study. If an event had taken place resulting in a difference of visual acuity (the criterion for an inter-ocular visual acuity difference was set at >0.2 logMar, 9 patients), the eye with the lower visual acuity was called the affected eye.

The patients were asked to binocularly view a calibrated projected visual acuity chart (Topcon ACP 8) while wearing their best optical correction. The chart was set one or two lines larger than the binocular visual acuity. Six different grey filters with transmissions of 10%, 25%, 35%, 50%, 75%, and 90% were presented in front of the affected or less dominant eye. When looking at the projected acuity chart, patients were asked to indicate any difference when holding a grey filter in front of the affected or less dominant eye. Several filters in a random and repeated manner were presented to the patients. All patients reacted repeatedly in a constant and positive way to the same transmission grey filter; therefore, the patients chose subjectively the visually most comfortable filter when viewing binocularly. The grey filter contact lens was fitted according to the chosen transmission and to the standards of the profession. Patients without a perceptible improvement with any particular filter were not fitted with a contact lens and were excluded from this study.

The material used was a pigmented 67% water polymer, Filcon 2 by Contamac^©^, DK Fatt 30 and manufactured by Cantor and Nissel^©^. One patient was refitted with a PMMA (polymethyl methacrylate) contact lens, DK Fatt 0, manufactured at Oculenti^©^ contact lens laboratory. Grey filter contact lens wear was evaluated during follow up visits. All patients were asked about the visual effects of contact lens wear and their motivation to continue contact lens wear. Visual acuity, anterior segment, comfort and fit of the contact lens were evaluated and subjective visual performance with the grey filter contact lens was assessed with a questionnaire (***Table 2***).

## Results

Nineteen patients were included (***Table 1***), 10 men and nine women. One patient was lost to follow up (n = 18). Mean age of the patients was 51 years (SD = 10, range 30–68 years). Causes and visual complaints of the disturbed binocular performance are listed in ***Table 1***. The most common causes were retinal diseases (63% of patients). The patients reported wavy images or grey fields in the binocular image, headache, or a double image. An inter-ocular visual acuity difference ≥ 0.2 logMar occurred in half (52%) of the patients. One patient had a large inter-ocular visual acuity difference due to amblyopia with double vision after anti-suppression therapy. Seven patients did not use any refractive correction, and eight patients wore glasses. The other four patients were habitual contact lens wearers, two with soft and two with rigid gas permeable contact lenses.

**Table 1:** Overview of patients’ characteristics. VA = Visual acuity. Stop = the reason for discontinuation of grey filter contact lens wear: A = no discontinuation; B = discontinuation, complaints solved; C = discontinuation, handling problems; and D = discontinuation, no effect on visual problems. Brvo = branch retinal vein occlusion.


NO	SEX	AGE START	OD/OS	TRANSMISSION	STOP	EFFECT	LENGTH WORN (MONTHS)	DIAGNOSIS	VISUAL COMPLAINT	VA LOGMAR OD	VA LOGMAR OS	VA DIFF.

1	F	39	OS	50	A	Yes	5	macular degeneration	grey field	0.00	0.40	0.40

2	M	58	OS	35	A	Yes	5	macular degeneration	wavy image	0.00	0.10	0.10

3	M	68	OS	35	A	Yes	6	maculopathy	wavy image	0.10	0.10	0.00

4	M	56	OD	50	A	Yes	19	optic neuritis	grey field	1.00	0.15	0.85

5	F	45	OS	50	A	Yes	46	intractable diplopia	double image	0.00	0.10	0.10

6	M	64	OS	50	A	Yes	5	intractable diplopia	double image	0.22	0.10	0.12

7	M	51	OS	75	A	Yes	6	trauma capitis	head-ache	0.05	0.70	0.65

8	M	57	OS	75	B	Yes	12	retinal detachment	grey field	–0.11	0.40	0.51

9	F	53	OD	25	B	Yes	17	retinal detachment	double image	1.30	0.00	1.30

10	F	55	OD	75	B	Yes	8	brvo	grey field	0.22	–0.08	0.30

11	F	58	OD	35	B	Yes	15	brvo	grey field	1.00	–0.08	1.08

12	M	48	OD	50	B	Yes	5	optic neuritis	grey field	1.30	–0.08	1.38

13	F	60	OS	35	C	No	1	macular degeneration	wavy image	0.00	0.52	0.52

14	M	50	OD	50	C	No	4	retinal detachment	wavy image	1.00	0.00	1.00

15	F	33	OS	25	D	No	4	optic neuritis	grey field	–0.08	–0.08	0.00

16	F	63	OD	75	D	No	5	N/A	head-ache	0.70	0.30	0.40

17	M	30	OD	50	D	No	15	intractable diplopia	double image	–0.04	–0.04	0.00

18	M	45	OD	75	D	No	1	intractable diplopia	double image	0.70	0.00	0.70

19	F	42	OS	50	N/A	N/A	N/A	trauma capitis	head-ache	–0.08	0.70	0.78


**Table 2:** Questionnaire.


Wear modality	How long (months) do you wear the contact lens?

	Do you wear the contact lens every day?

	How many hours per day?

Effect of the contact lens in daily life	Did you discontinue wearing the contact lens?

	If you discontinued wearing the contact lens, why?

	disappearance of visual problems

	the contact lens had no effect on visual quality in daily life

	handling contact lens

	comfort contact lens

	compliance contact lens

	changes to the other eye

	other …..

Contact lens wear	If the visual complaints are still present, but you discontinued the contact lens wear, did you take any action to diminish these complaints?


**Figure 1:**
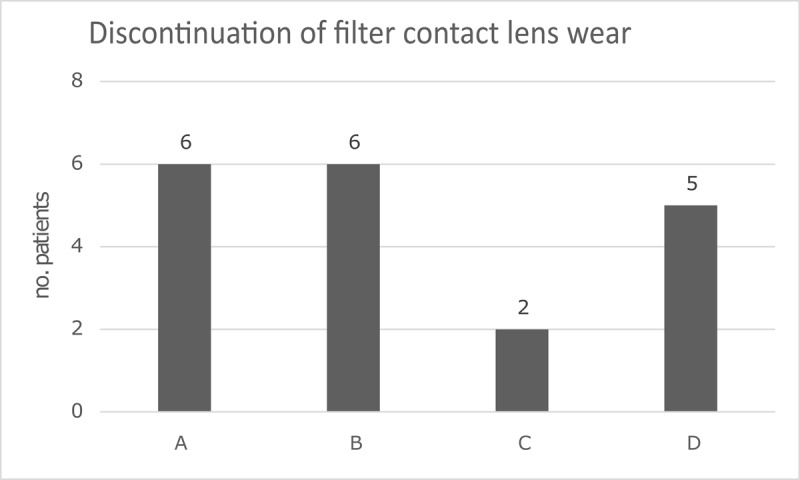
Discontinuation of filter contact lens wear.

Twelve patients (67%) reported good results when wearing the grey filter contact lens. Of this group, seven patients continued to wear the grey filter contact lens. These patients comprise Group A (***Table 1*** and ***Figure 1***). Five patients of this group discontinued the filter contact lens, because their visual complaints disappeared during filter contact lens wear and remained absent after contact lens wear was terminated. These patients comprise Group B (***Table 1*** and ***Figure 1***). Two patients (11%) were not able to handle the contact lens and discontinued the wear of the filter contact lens. These patients comprise Group C (***Table 1*** and ***Figure 1***). Despite the positive effect when testing the grey filters, the effect in daily life was found to be disappointing by four patients (22%), designated Group D, and stopped treatment because the filter contact lens did not resolve their visual problems (***Table 1*** and ***Figure 1***).

The filter with 50% transmission was chosen by most patients (40%), and no patients chose the filters with the least (10%) or most (90%) transmission. The contact lens was used for a median of 5.5 months (range 1–46 months). The mean inter-ocular difference in logMAR visual acuity between both eyes was –0.20 ± 0.65.

The inter-ocular difference in visual acuity did not significantly influence the choice of filter (p = 0.956; Krukas Wallis) or the success of the treatment (p = 0.509; Mann Whitney).

No correlation was found between the inter-ocular difference in visual acuity and the duration of contact lens wear in months (r = –0.019, p = 0.939; Spearman’s rank correlation). Patients who showed an effect of treatment wore the contact lens longer (median 7 months, range 5–46 months) compared to those who did not show an effect (median 4 months, range 1–15) (p = 0.016, Mann Whitney). The intensity of the filter used did not differ between the two groups (p = 0.964 Mann Whitney).

## Discussion

In this study, 12 patients (67%) were considered to have successful outcomes. No significant correlation was found between success and the choice of filter transmission, age, or inter-ocular visual acuity. The group of five patients (Group B) who were cured of their binocular visual complaints during grey filter contact lens wear and discontinued the grey filter contact lens is a heterogeneous group. The only similarity is the monocular visual acuity loss, with a difference of more than 0.2 logMAR, due to pathology. There is no similarity in transmission of the filter or duration of contact lens wear. Possibly the brain adapted and learned to ignore the image formed in the affected eye, unless this image was needed (e.g., use of the peripheral visual field of the affected eye or if the unaffected eye was closed). The filter contact lens could have played a role in this adaptation and improved function, secondary to neuro-adaptive processes. However, the structure of this study does not allow any evidence for this argument and more research is needed to understand how and why this improvement occurred.

The starting point of this option was the hypothesis that a response to a stimulus was not seen with visual evoked potentials (VEP) when the second stimulus was delayed, but not more than 40 msec. (Riemslag, Spekreijse & Van Wessem 1985). This could offer a solution for patients who insufficiently adapted to an acutely arising difference in binocular performance between both eyes, leading to problems with binocular visual functioning. We chose to use a contact lens with grey filter over grey spectacle glasses because the contact lens influences all light entering the eye. The use of a contact lens to induce artificial central suppression is a theoretically viable option, particularly in patients with impaired central fusion (for example, macular disease or central fusion disruption) but preserved peripheral fusion, as this may allow the eyes to continue working together. It may also be advantageous in cases where peripheral fusion is not present, but preservation of the peripheral visual field would support mobility and spatial awareness. The exact mechanism on how the grey filter contact lens works has to be subject in future research.

Acquired diminished binocular visual performance is a difficult problem to treat. If causative treatment is no option, the only option is to ignore a disturbing image of an affected eye. Some patients manage to ignore this image, but other patients cannot. For the patients without the ability to ignore the disturbing image, there are different options available, but all block or hamper a part or the whole field of vision. Although some patients experience a successful improvement with these options, other patients continue to suffer from the acquired diminished binocular visual performance. Blocking a part of the visual field leads to difficulty with driving, stumbling onto persons on the side of the affected eye, or even feeling nauseous. This is the disadvantage of every option, also with the scotogenic contact lens. To offer an acceptable alternative to these patients, the other option with the grey filter contact lens was investigated.

Many types of solutions to overcome acquired reduced binocular visual performance have been suggested as described in the introduction. The relative rareness of the pathology, and the varied solutions published, make it difficult to propose a standardized solution. One solution is to occlude the affected eye in order to completely eliminate the central and peripheral images. No studies were found using the eye-patch, and just a few studies (Astin et al. 1998; Newsham et al. 2018) were identified that looked at occlusive contact lenses. Newsham and colleagues (2018) have used occlusive contact lenses for 10 patients. In four patients this was used as first line treatment, and it was overall successful in five patients. Astin and colleagues (1998) described four successful case reports with occlusive contact lenses.

In the UK a large survey has been held among ophthalmologists regarding opaque IOLs (Kwok & Watts 2009). An opaque IOL was implanted in 46 patients and was successful in 31 patients (including 9 patients who needed pilocarpine for miosis because of light transmission around the IOL optic). Newsham and colleagues (2018) reported a successful outcome in six out of seven patients implanted with an opaque IOL.

A second possible solution is to diminish the complaints and try to preserve peripheral vision as much as possible. Most studies investigating this solution use Bangerter filters (De Pool et al. 2005; Iacobucci, Furr & Archer 2009; McIntyre & Fells 1996; Newsham, O’Connor & Harrad 2018; Rutstein 2010; Silverberg et al. 1999). The success rate with Bangerter filters is inconclusive. There were initial measurements with the Bangerter filters in the studies of De Pool and colleagues (2005) and Rutstein (2010), but follow up was not recorded. McIntyre and colleagues (1996) gave diverse criteria to evaluate the efficiency of Bangerter filters and reported seven unsatisfactory outcomes in 20 patients. Silverberg and colleagues (1999) reported seven patients in whom the diplopia was eliminated, with two unsuccessful in daily life. Newsham and colleagues (2018) described three patients who used a Bangerter filter as a first option of treatment, but the treatment failed in two patients. Iacobucci and colleagues (2009) reported three out of three successful cases with Bangerter filters in combination with prism correction. It has been generally advised to use the weakest density to just eliminate the image of the affected or less dominant eye to allow as much peripheral vision as possible (Rutstein 2010). Even so, the peripheral field of vision is blurred with Bangerter filters.

A third option to eliminate the image of the affected eye is opaque tape. The results are variable. De Pool and colleagues (2005) reported 46 of 58 patients showed effective resolution of symptoms and were comfortable with the tape, while Newsham and colleagues (2018) reported no success with all four of their patients. Politzer (1996) described two successful case reports with a spot patch opaque tape in the line of sight of the affected eye. One might ask whether this is a cosmetic justified option.

Other solutions in the literature are scarce, with few patients treated and reported. One such solution is a scotogenic contact lens (Robert et al. 2015). The contact lens was introduced, but no further results have been reported to date. Newsham and colleagues (2018) reported a defocus with a high-powered lens in one patient, without success. There is one case report describing one patient in whom diplopia was eliminated with a central corneal tattoo (Laria, Alio & Pinero 2010). Patients with prolonged wearing time of the grey filter contact lens after the questionnaire reported no negative remarks about the peripheral vision and mentioned a better quality of life with the use of the grey filter contact lens. In this retrospective study we could not report any findings about the quality of life before and after the use of the grey filter contact lens, because this was not noted in the files. The exact impact on the peripheral vision and measuring the quality of life with, for instance, ASQE (Felius et al. 2007) has to be investigated in future research. A randomized controlled trial comparing the effect of Bangerter filters and the grey filter lens would be interesting, the peripheral vision and the quality of live included in such a study. The main disadvantage of the grey filter contact lens in one eye is a disturbed cosmetic appearance. This disadvantage is also present with other options to treat disturbing binocular diplopia. If we can diminish the grey filter part to the central 6–8 mms of the contact lens, the cosmetic appearance is less disruptive. On the contrary, some patients mentioned the darker color of the eye as an advantage because of being identified with a perceptible eye problem.

In conclusion, the grey filter contact lens can be considered to be a good treatment option to diminish complaints of reduced binocular visual performance after unilateral acquired reduced visual performance, if there is no further causative/causal therapy possible. It is a non-invasive, easily reversible treatment that does not impede the field of vision.
